# Editorial: Molecular pathways and signaling molecules in cancer therapy: advances and innovations

**DOI:** 10.3389/fimmu.2026.1924399

**Published:** 2026-07-14

**Authors:** Abdullah Farhan ul Haque Saeed, Sherine Elsawa, Chamini Perera

**Affiliations:** 1 Department of Internal Medicine, University of Michigan, Ann, Arbor, MI, United States; 2 Department of Molecular, Cellular, and Biomedical Sciences, University of New Hampshire, Durham, NH, United States; 3 School of Clinical Medicine, Faculty of Medicine and Health, University of New South Wales, Sydney, NSW, Australia

**Keywords:** cancer resistance, cancer therapy, immunotherapy, molecular signaling, molecular pathways, onco-immunology

Cancer remains a leading cause of mortality worldwide, driven by diverse molecular alterations and a dynamic tumor microenvironment (TME) that together fuel progression and therapeutic resistance (1, 2). Molecular pathways and signaling molecules sit at the heart of cancer initiation, progression, and therapeutic resistance, making them prime targets for precision oncology. Dysregulated cascades such as PI3K/AKT/mTOR, RAS/RAF/MEK/ERK, and p53-centered stress networks integrate oncogenic mutations, epigenetic alterations, and microenvironmental cues to drive unchecked proliferation, survival, and metastatic dissemination (3, 4). Despite major advances with kinase inhibitors and immune checkpoint blockade, inter- and intra-tumoral heterogeneity, pathway crosstalk, and adaptive rewiring frequently blunt responses and foster resistance, highlighting the need for a deeper, systems-level understanding of signaling architecture across tumor and stromal compartments (5–7). Against this backdrop, this Topic, “Molecular Pathways and Signaling Molecules in Cancer Therapy: Advances and Innovations,” is timely because therapeutic success increasingly depends on our ability to map, model, and selectively perturb these networks rather than individual nodes.

**Figure 1:**
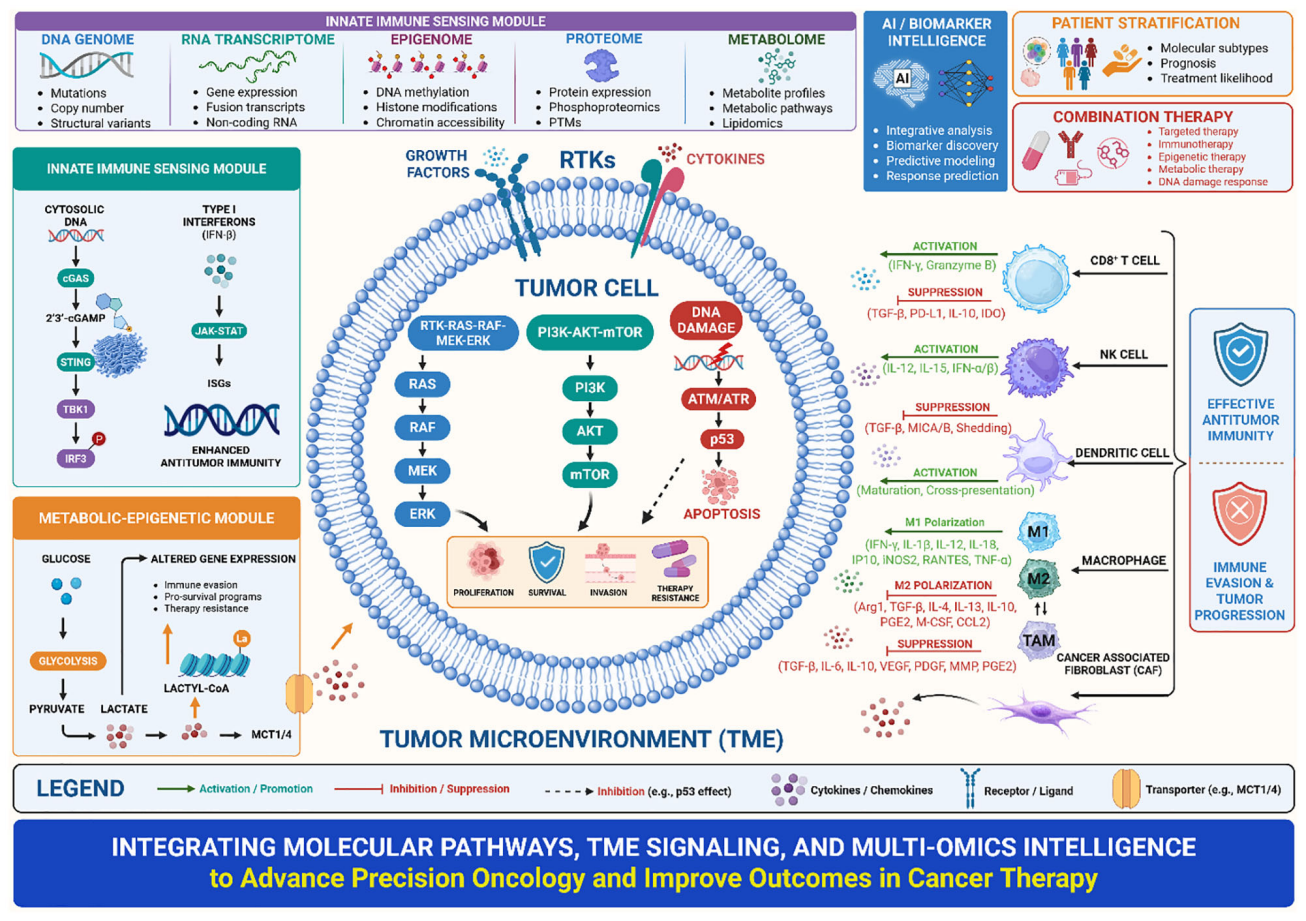
Signaling networks coordinate tumor development, immune evasion, and therapy resistance to drive cancer progression. RTK/RAS/RAF/MEK/ERK and PI3K/AKT/mTOR activation enhances tumor cell proliferation and survival, but p53-dependent DNA damage responses are disrupted, removing a key barrier to malignancy. CD8^+^ T cells, NK cells, dendritic cells, macrophages, and fibroblasts form the tumor microenvironment through cytokines, checkpoint signaling, and stromal interactions that may boost or suppress antitumor immunity. STING-dependent type I interferon signaling from cytosolic DNA activates dendritic cells and primes T cells, adding another layer of control. While metabolic reprogramming towards glycolysis increases lactate generation, lactate-driven lactylation modifies gene expression to favor immune evasion, angiogenesis, and tumor plasticity. Integrated multi-omics analysis of DNA, RNA, epigenomic, proteomic, and metabolomic changes helps find biomarkers, stratify patients, and guide sensible combination therapy.

Recent studies underscore that oncogenic signaling cannot be viewed in isolation from innate immunity, metabolism, and the TME, further elevating the importance of this Topic. Innate sensing via the cGAS-STING pathway, for example, has emerged as a critical determinant of antitumor immunity and a promising axis for drug development, yet exhibits context-dependent pro- and anti-tumor roles that demand careful mechanistic dissection (8, 9). Parallel advances in multi-omics profiling are resolving how transcriptional, epigenetic, and metabolic rewiring, such as lactate-driven lactylation, shape immune evasion and therapeutic resistance across metastatic niches. Likewise, integrative, AI- and machine-learning-enabled analysis of these datasets is beginning to yield pathway-level biomarkers that can guide immunotherapy selection, rational combinations, and dynamic regulation in addition to other similar mechanisms that shape the TME (10–12). Collectively, these developments frame molecular pathways and signaling molecules not merely as mechanistic curiosities but as the organizing principle for next-generation cancer therapies (13) (**Figure 1**).

This Research Topic was envisioned to bring together mechanistic and translational insights into how signal transduction, metabolism, and immune crosstalk can be leveraged to design more precise and durable cancer therapies. The topic emphasizes that cancer therapies increasingly succeed or fail based on our ability to understand and manipulate dysregulated pathways, such as PI3K/AKT/mTOR, RAS/RAF/MEK/ERK, and p53 networks, and their integration with the tumor immune microenvironment and the stromal compartment (14–16). Traditional cytotoxic approaches are frequently undermined by intrinsic heterogeneity and resistance acquisition, underscoring the need for pathway-targeted agents, immunotherapies, and rational combinations guided by molecular biomarkers (17, 18). The contributions gathered here span comprehensive reviews and original research, collectively illuminating how innate immune sensing, metabolic rewiring, and multi-omics discovery can be translated into actionable therapeutic strategies. This editorial highlights several anchor articles that exemplify the Topic’s central themes and are poised to guide future work in pathway-directed and immune-based cancer therapy.

A cornerstone of this Research Topic is the review by Wang et al. This article synthesizes rapidly expanding understanding of the cyclic GMP-AMP synthase (cGAS)-stimulator of interferon genes (STING) pathway as a central sensor of cytosolic DNA that orchestrates type I interferon responses at the interface of tumor immunity, autoimmunity, and sterile inflammation. The authors describe how cGAS-STING activation can exert both tumor-suppressive and context-dependent protumor effects through diverse effector outputs, including interferons, inflammatory cytokines, and immunogenic cell death (19).

Importantly, Wang et al. document emerging small-molecule agonists and other agents designed to activate cGAS-STING in cancers, summarizing preclinical efficacy, mechanisms of action, and current limitations. They highlight challenges such as dose-limiting systemic inflammation, variable STING expression across tumor types, and the need to integrate pathway activation with checkpoint blockade or other immunomodulatory strategies. By providing a mechanistic blueprint and therapeutic landscape, this Review positions the cGAS-STING axis as a prototypical example of how innate immune signaling can be rationally harnessed in precision oncology, exactly aligned with the aims of this Topic (19).

Another flagship contribution is the review by Yang et al., which reframes lactate from a metabolic waste product to a pleiotropic fuel, signaling molecule, and epigenetic substrate. Drawing on biochemical and immunologic data, the authors explain how lysine lactylation integrates glycolytic reprogramming with transcriptional control, thereby influencing tumor plasticity, immune suppression, and therapy resistance. They synthesize evidence that lactylation of histones and non-histone proteins modulates pathways governing proliferation, angiogenesis, and immune evasion within the TME (20).

Significantly, the review surveys therapeutic approaches targeting lactate production and transport, as well as enzymes that write or erase lactylation, to recondition the TME and restore antitumor immunity. This includes inhibitors of lactate dehydrogenase, monocarboxylate transporters, and emerging strategies that combine metabolic interventions with immunotherapy. By framing lactylation as a “metabolic language” that tumors use to communicate with stromal and immune cells, the authors provide a conceptual scaffold for future interventions that jointly target metabolism and signaling, echoing the Topic’s focus on pathway-level innovation.

Moreover, several contributions dissect how pathway dysregulation manifests in specific malignancies and how multi-omics can uncover actionable targets. Shebbo et al. reviewed the heterogeneous molecular architecture of oral squamous cell carcinoma (OSCC). They summarize key oncogenic cascades-including EGFR and related receptor tyrosine kinases, PI3K/AKT/mTOR, and cell-cycle regulators-alongside genetic susceptibility loci and microbial influences that together contribute to OSCC’s poor 5-year survival. The review critically assesses why many emerging targeted and immunotherapies have failed to yield durable benefit in OSCC and argues for integrative approaches that combine pathway inhibition, immune modulation, and improved patient stratification (21).

Complementing this disease-focused perspective, Zheng and Xu offer an original contribution. Using Mendelian randomization, epigenome-wide association, methylation QTL mapping, and eQTL integration, they identify omega-3-related CpG sites and downstream targets that causally link circulating metabolites, CD4^+^ T-cell traits, and colorectal cancer (CRC) risk. The study converges on SLC6A19 as a candidate inhibitory target whose expression in CD4^+^ T cells, colonic epithelium, and CRC cells associates with prognosis and immune infiltration. Functional assays demonstrate that SLC6A19 overexpression suppresses CRC cell proliferation, migration, invasion, and xenograft tumor growth, suggesting a tractable node in a metabolism-immune-tumor axis (22).

Together, these articles illustrate how deep multi-omic interrogation can move beyond descriptive pathway maps to nominate and validate specific transporters and signaling molecules as therapeutic entry points in defined tumor contexts. They embody the Topic’s commitment to linking molecular mechanisms with translational opportunities across diverse cancer types.

A recurring theme in this Research Topic is the quest for biomarkers and cell-intrinsic programs that can refine patient selection and inform combination strategies with immunotherapy. In a study by Poddubskaya et al., the authors summarize current and emerging markers, ranging from PD-L1 expression and tumor mutational burden to gene-expression signatures and components of the TME that modulate response to PD-1/PD-L1 blockade in non-small cell lung cancer (NSCLC). They evaluate the strengths and limitations of each biomarker class, highlighting issues of assay standardization, spatial heterogeneity, and dynamic evolution under therapy. The authors advocate for composite, multi-parameter models that integrate genomic, transcriptomic, and immunologic features, a concept echoed by several other contributions on this Topic (23).

Adding another mechanistic layer, a contribution by Zang et al. focuses on disulfidptosis, a recently described form of regulated cell death triggered by disulfide stress under conditions of high SLC7A11-mediated cystine uptake and glucose starvation. The authors develop disulfidptosis-related gene signatures that stratify patients prognostically and correlate with immune infiltration patterns, thereby linking metabolic vulnerability to immunologic context. They discuss how therapies that induce or exploit disulfidptosis might synergize with checkpoint blockade or other immunotherapies by reshaping the TME and exposing neoantigens (24).

In addition, translational relevance is underscored by the case-based contribution. Hao et al. report JAK1/2 inhibition in a refractory natural killer large granular lymphocytic leukemia. By detailing the molecular rationale, clinical course, and immunologic consequences of golidocitinib treatment, the authors illustrate how pathway-targeted agents can be repurposed for rare, pathway-defined malignancies when guided by genomic and signaling insights. This contribution exemplifies how precision medicine at the single-patient level can inform broader strategies for targeting JAK–STAT and related signaling networks in hematologic cancers (25).

Across these articles, several convergent themes emerge. First, cancer signaling must be understood as a multi-layered network spanning innate sensing (cGAS–STING), metabolic and epigenetic rewiring (lactylation, disulfidptosis), and immune–stromal crosstalk (T-cell traits, TME composition) rather than isolated pathways. Second, integrative multi-omics and functional validation are essential to move from associative biomarkers to bona fide therapeutic targets, as illustrated by SLC6A19 in CRC and disulfidptosis signatures across solid tumors. Third, translation to the clinic will require careful attention to toxicity, context-specific effects, and rational combination strategies-particularly when activating innate immunity or perturbing core metabolic circuits.

Looking ahead, the work collected in this Topic points toward several priorities. These include mapping pathway activity and metabolic states at single-cell and spatial resolution to capture intratumoral heterogeneity; embedding pathway-level biomarkers into clinical trial design; and developing therapeutic regimens that concurrently target tumor-intrinsic signaling, metabolic dependencies, and immune checkpoints. By assembling contributions that span conceptual reviews, disease-focused syntheses, and multi-omics original research, *Molecular Pathways and Signaling Molecules in Cancer Therapy: Advances and Innovations* provides a foundation for the next generation of mechanism-guided, immune-competent cancer therapies.
